# Intestinal Organoids in Colitis Research: Focusing on Variability and Cryopreservation

**DOI:** 10.1155/2021/9041423

**Published:** 2021-09-17

**Authors:** Talke F. zur Bruegge, Andrea Liese, Sören Donath, Stefan Kalies, Maike Kosanke, Oliver Dittrich-Breiholz, Sandra Czech, Verena N. Bauer, André Bleich, Manuela Buettner

**Affiliations:** ^1^Institute for Laboratory Animal Science, Hannover Medical School, Hannover, Germany; ^2^Institute of Quantum Optics, Leibniz University Hannover, Hannover, Germany; ^3^Lower Saxony Center for Biomedical Engineering, Implant Research and Development (NIFE), Hannover, Germany; ^4^Research Core Unit Genomics, Hannover Medical School, 30625 Hannover, Germany

## Abstract

In recent years, stem cell-derived organoids have become a cell culture standard that is widely used for studying various scientific issues that were previously investigated through animal experiments and using common tumor cell lines. After their initial hype, concerns regarding their standardization have been raised. Here, we aim to provide some insights into our experience in standardizing murine colonic epithelial organoids, which we use as a replacement method for research on inflammatory bowel disease. Considering good scientific practice, we examined various factors that might challenge the design and outcome of experiments using these organoids. First, to analyze the impact of antibiotics/antimycotics, we performed kinetic experiments using ZellShield® and measured the gene expression levels of the tight junction markers *Ocln*, *Zo-1*, and *Cldn4*, the proliferation marker *Ki67*, and the proinflammatory cytokine *Tnfα*. Because we found no differences between cultivations with and without ZellShield®, we then performed infection experiments using the probiotic *Escherichia coli* Nissle 1917 as an already established model setup to analyze the impact of technical, interexperimental, and biologic replicates. We demonstrate that interexperimental differences pose the greatest challenge for reproducibility and explain our strategies for addressing these differences. Additionally, we conducted infection experiments using freshly isolated and cryopreserved/thawed organoids and found that cryopreservation influenced the experimental outcome during early passages. Formerly cryopreserved colonoids exhibited a premature appearance and a higher proinflammatory response to bacterial stimulation. Therefore, we recommend analyzing the growth characteristics and reliability of cryopreserved organoids before to their use in experiments together with conducting several independent experiments under standardized conditions. Taken together, our findings demonstrate that organoid culture, if standardized, constitutes a good tool for reducing the need for animal experiments and might further improve our understanding of, for example, the role of epithelial cells in inflammatory bowel disease development.

## 1. Introduction

Over the past decade, stem cell-derived organoid culture is a well-known system that has evolved from an exciting new tool for investigating scientific issues to a standard cell culture and *in vitro* method. Organoids were initially introduced as a promising model for basic research on disease development and progression, toxicological drug testing, and regenerative medicine [1–5]. Much progress has been made, for example, generating organoid structures from many different origins and establishing various protocols for all types of applications. There was much hype on organoids when they were first introduced, but some skepticism regarding their standardization combined with experimental considerations has recently emerged [5–7]. However, since their introduction, the definition of organoids has been agreed upon, and most scientists are currently aware of organoids [8] and their classification [9]. Specifically, organoids are 3D structures generated from pluripotent stem cells, such as iPSCs or ESCs, or from tissue-resident neonatal or adult stem or progenitor cells that are cultured in a tissue-like extracellular matrix (ECM). In the presence of niche and growth factors, these cells differentiate into all functionally relevant cell types and spontaneously self-assemble into 3D structures that can perform some of the donor organ's functions [1–3, 10].

In our research group, we mainly focus on intestinal epithelial organoids from the murine colon, hereafter also referred to as colonoids [9]. According to the 3R principles, we use these colonoids as a replacement tool for investigating the pathomechanism of inflammatory bowel disease (IBD) instead of performing *in vivo* studies. IBD is a multifactorial disease that depends on various factors, such as genetic predisposition, environmental factors, and alterations in the microbial gut flora [11]. Many *in vivo* models that exhibit genetic predisposition have been established, for example, the *Il10*-knockout mouse strain [12]. However, environmental and microbial factors are more difficult to display due to marked differences between the mouse model and the human situation. Furthermore, current *in vitro* models using, for example, Caco-2 cells often lack the physiological properties of a human tissue. Therefore, Dotti and Salas [6] reviewed the usage of *ex vivo* human intestinal organoids for research on IBD and judged them to be a suitable tool for analyzing disease mechanisms, although methods for their standardization are needed.

This recommendation is in accordance with the claims made by another research group [5], who states that the general reproducibility of organoid cultures is essential for their use, e.g., in developmental and drug testing studies. It is also important to consider the scalability and safety of organoids when attempting to use these systems in human regenerative medicine [5].

What we learned over the last few years in our organoid research is that the pace of experimental progress is rather slow due to the need to understand the fundamental basis of organoid formation and its requirements. As also stated by Huch et al. [5], we can only exhibit progress by “carefully laying the groundwork” for creating a useful product and facing the hype of organoid research with realistic expectations. As commented by Spence [7], organoids lack a commonly well-accepted standard regarding their use in experiments, and every laboratory using organoids has established its own standardizing methods.

This paper is aimed at collecting several strategies for standardizing 3D organoids to increase their value for the study of various scientific issues. According to good scientific practice, we want to note important aspects that should be considered in experimental designs to render 3D organoids a more predictable and reliable tool, for instance, regarding their technical, interexperimental, and biologic replicability. Additionally, we provide the first demonstration that the cryopreservation of intestinal epithelial organoids might influence the experimental outcome and should thus be analyzed in preliminary tests.

## 2. Materials and Methods

### 2.1. Animals

All experiments in this study were conducted in accordance with the European Directive 2010/63/EU [13] and German animal protection laws and were approved by the Local Institutional Animal Care (File: 2015/78). Male, 9- to 12-week-old C57BL/6J (wild-type) mice were obtained from the Central Animal Facility (Hannover Medical School, Hannover, Germany), where they were formerly housed in individually ventilated cage systems under standardized room and specific pathogen-free conditions according to the recommendations of the Federation of European Laboratory Animal Science Association [14]. Routine microbiological monitoring did not reveal any evidence of infection with common murine pathogens with the exception of [*Pasteurella*] *pneumotropica*, *Staphylococcus aureus*, *Klebsiella oxytoca*, and *Helicobacter* sp.

Prior to this study, we carefully timed and interweaved all of our experiments to ensure the use of the minimum number of animals for the maximum number of experiments. In total, 17 animals were sacrificed to obtain the 17 independent colonoid lines that were used in this study. All generated colonoid lines were cryopreserved and can be reused in further studies. For reduction reasons, most of the data of the freshly isolated colonoid group (see Section 2.9) were obtained from the analysis of technical and interexperimental replicability and biological variability (see Section 2.8); therefore, the data are repetitively used in the various experiments.

### 2.2. Preparation of Organoids

The isolation and plating of crypts were performed as already published by us in Brooks/zur Bruegge et al. [15] with the following alterations/specifications: After transfer into dissociation buffer (DPBS containing 54.9 mM sorbitol and 43.4 mM sucrose), the colonic tissue pieces were thoroughly mixed by hand until the suspension became turbid with detached crypts. The crypt suspension was filtered (70 *μ*m pores) and centrifuged, and the pellet was resuspended in Matrigel® (Corning™, New York, NY, USA) and organoid growth medium (DMEM [high glucose, pyruvate, GlutaMAX™] [Thermo Fisher Scientific, Waltham, USA] with 50% L-WRN supernatant [ATCC® CRL3276™ in DMEM [high glucose, pyruvate, GlutaMAX™] plus 10% fetal calf serum [FCS]] supplemented with 10% FCS [total concentration], 1× B27 [Invitrogen, Carlsbad, CA, USA], 1× N_2_ [Invitrogen, Carlsbad, CA, USA], 10 *μ*M Y-27632 [Tocris, Bristol, UK], 50 ng/*μ*L recombinant mouse epidermal growth factor [Sigma-Aldrich, St. Louis, MO, USA], and 1× ZellShield® [Biochrom, Berlin, Germany]) in a ratio of two-thirds to one-third, respectively. Under continuous mixing, 50 *μ*L of the Matrigel®-and-crypt mixture was pipetted into wells of a 24-well plate in alternating diagonal rows. For a better nutrient distribution throughout the gel, the droplets were mechanically flattened with the pipette tip, polymerized at 37°C for 30 minutes and overlaid with 500 *μ*L of organoid growth medium. Organoids were cultured with 5% CO_2_ at 37°C, and the medium was changed every 3-4 days unless stated otherwise.

### 2.3. Passaging of Organoids

The organoids were passaged weekly unless stated otherwise. For each passage, the Matrigel® droplets were dissolved by thorough pipetting after the addition of ice-cold DPBS, and the organoids were split through a 27G 1/2″ cannula. The crypt suspension was centrifuged, and the pellet was resuspended in fresh Matrigel® and organoid growth medium and processed as described above (Section 2.2).

### 2.4. Cryopreservation and Thawing of Organoids

Freshly isolated organoids were grown for 1 week until passage 1 and then processed as described in Section 2.3, titled “Passaging of Organoids.” Instead of Matrigel®, the pellet was resuspended in FCS with 10% DMSO and then frozen at -20°C in a Mr. Frosty™ (Fisher Scientific GmbH, Schwerte). After 24 h, the Mr. Frosty™ was transferred to -80°C, and 24 h later, the cryovials were transferred to liquid nitrogen until further use.

The cryopreserved organoids used for infection experiments were rapidly thawed at 37°C until the suspension became liquid. The organoids were then immediately transferred into ice-cold DPBS with 10% FCS, centrifuged, and plated as described in Section 2.2, titled “Preparation of Organoids.”

### 2.5. Effects of ZellShield® on Organoid Kinetics

The organoids used to assess the effects of ZellShield® on organoid kinetics were isolated and cultivated as mentioned above (Sections 2.2 and 2.3) over 3 weeks in the presence of ZellShield® until passage 3. The organoids from passage 3 were then cultivated for 10 days in the presence of ZellShield®, and the medium was changed every 3 days, with the last change occurring one day prior to the experiment. On day 10, the old organoid growth medium was replaced by fresh organoid growth medium with or without ZellShield®, and the organoids were cultured for 1, 2, 4, 6, and 12 hours at 37°C in the presence of 5% CO_2_. After incubation, the supernatant was removed and stored at -20°C. The plate was immediately placed on ice, the Matrigel® was dissolved, and the organoid structures were disrupted by thorough pipetting after the addition of ice-cold DPBS. The suspension was centrifuged, and the pellet was resuspended in RNA Quick-RNA™ Micro Prep Kit Lysis Buffer (Zymo Research, Irvine, CA, USA) and stored at -80°C until further processing for quantitative real-time PCR (qPCR) analysis. Two wells of each condition were pooled to obtain the supernatant and lysed samples. The whole experimental setup from isolation to sample collection was repeated in five independent experiments.

### 2.6. Cultivation of *E. coli* Nissle 1917

The *E. coli* Nissle 1917 strain (*Ec*N) for the infection experiments was cultivated as already published by us in Brooks/zur Bruegge et al. [15].

### 2.7. *Ec*N Infection Experiments

The organoids used in the *Ec*N infection experiments were cultivated as described in Section 2.5, titled “Effects of ZellShield® on Organoid Kinetics”; again, organoids at passage 3 were used on day 10 for the infection. The old growth medium was exchanged with fresh medium without ZellShield® to obtain the control samples, and the infection samples were administered the bacterial suspension at 1 : 25 dilution in organoid growth medium without ZellShield®. The control and infection samples were incubated for 1 hour at 37°C in 5% CO_2_. After incubation, all supernatants and lysed organoid samples were collected and stored using the protocol described in Section 2.5.

### 2.8. Technical and Interexperimental Replicates and Biological Variability

For the analysis of technical and interexperimental differences and biological variability, we performed five independent *Ec*N infection experiments as described above (Sections 2.6 and 2.7) on five different days using three different biological replicates (organoid lines) per experiment and three technical replicates (two wells pooled to prepare each sample) per organoid line. In total, we used 15 independent organoid lines for this experiment.

### 2.9. Freshly Isolated vs. Cryopreserved Organoids

For the comparison between freshly isolated and cryopreserved organoids, we performed seven independent *Ec*N infection experiments according to the experimental setup described above (Sections 2.6 and 2.7) using both types of organoids. The data from 15 freshly isolated organoid lines were obtained from the experiments described in Section 2.8, titled “Technical and Interexperimental Replicates and Biological Variability,” and were repetitively used in this context. In addition, two additional freshly isolated organoid lines were generated as a comparison group for the last cryopreserved batches. The 12 cryopreserved organoid groups were thawed as described in Section 2.4, titled “Cryopreservation and Thawing of Organoids,” at passage 1 one week after the corresponding freshly isolated organoid groups that were simultaneously infected; therefore, the same passage was used for both conditions.

### 2.10. RNA Isolation and cDNA Synthesis

To quantify the gene expression levels in the collected lysis samples, intracellular RNA was isolated using the RNA Quick-RNA™ Micro Prep Kit (Zymo Research, Irvine, CA, USA), and up to 1 *μ*g of RNA was transcribed into cDNA using the QuantiTect® Reverse Transcription Kit (Qiagen®, Hilden, Germany) according to the manufacturer's instructions. Organoid samples for cDNA library generation and sequencing were lysed and processed according to the manufacturer's instructions using the RNeasy Mini Kit (Qiagen®, Hilden, Germany) with additional DNase digestion with the RNase-Free DNase Set (Qiagen®, Hilden, Germany) and stored at -80°C until further use.

### 2.11. Quantitative Real-Time PCR (qPCR)

The qPCR-based quantification of the gene expression levels of the cDNA samples was performed using a TaqMan®-based singleplex assay with *Actb* (Mm00607939_s1) as the endogenous control gene, *Mki67* (Mm01278617) and *Slc5a1* (Mm00451210_m1) as the target, and the following TaqMan®-based multiplex assays: 4-plex 1 [*Actb* (Mm00607939_s1_qsy_ABY) as the endogenous control gene, *Cldn2* (Mm00516703_s1_VIC; data not shown), *Cldn7* (Mm00516817_m1_qsy_JUN; data not shown), and *Tnfα* (Mm00443258_m1_FAM)] and 4-plex 2 [*Cldn4* (Mm_00515514_s1_qsy_ABY), *Cldn8* (Mm00516972_s1_qsy_JUN; data not shown), *Ocln* (Mm00500912_m1_FAM), and *Tjp1* (Mm01320638_m1_VIC)] (all from Thermo Fisher Scientific, Waltham, MA, USA). SYBR® Green-based QuantiTect Primer Assays (Qiagen®, Hilden, Germany) were used for *Actb* (Mm_Actb_1_SG) as the endogenous control gene, and *Chga1* (Mm_Chga_1_SG) and *Muc2* (Mm_Muc2_2_SG) as target genes. Each sample was either measured in duplicate or triplicate using a QuantStudio™ 6 Flex Real-Time PCR System (Applied Biosystems™, Foster City, CA, USA). Relative quantification (RQ) was performed using the 2^−*ΔΔC*T^ method [16].

### 2.12. Library Generation, Sequencing, and Raw Data Processing

Library Generation, Quality Control, and Quantification Were Performed as Described Previously [17]. 500 ng of total RNA per sample was utilized as input for mRNA enrichment procedure with “NEBNext® Poly(A) mRNA Magnetic Isolation Module” (E7490L; New England Biolabs) followed by stranded cDNA library generation using “NEBNext® Ultra II Directional RNA Library Prep Kit for Illumina” (E7760L; New England Biolabs). All steps were performed as recommended in user manual E7760 (Version 1.0_02-2017; NEB) except that all reactions were downscaled to 2/3 of initial volumes [17]. Furthermore, one additional purification step was introduced at the end of the standard procedure using 1× “Agencourt® AMPure® XP Beads” (#A63881; Beckman Coulter, Inc.) [17].

cDNA libraries were barcoded by dual indexing approach using “NEBNext Multiplex Oligos for Illumina–96 Unique Dual Index Primer Pairs” (6440S; New England Biolabs) [17]. All generated cDNA libraries were amplified with 7 cycles of final PCR.

Fragment length distribution of individual libraries was monitored using “Bioanalyzer High Sensitivity DNA Assay” (5067-4626; Agilent Technologies) [17]. Quantification of libraries was performed by use of the “Qubit® dsDNA HS Assay Kit” (Q32854; Thermo Fisher Scientific) [17].

#### 2.12.1. Library Denaturation and Sequencing Run

Equal molar amounts of 12 individually barcoded libraries were pooled for a sequencing run. The library pools were denatured with NaOH and were finally diluted to 1.8 pM according to the *Denature and Dilute Libraries Guide* (Document # 15048776 v02; Illumina) [17]. 1.3 mL of denatured pool was loaded on an Illumina NextSeq 550 sequencer using a High Output Flow Cell kit for 1 × 76 bp single reads (20024906; Illumina) [17]. Sequencing was performed with the following settings: sequence reads 1 and 2 with 38 bases each and index reads 1 and 2 with 8 bases each.

#### 2.12.2. BCL to FASTQ Conversion

BCL files were converted to FASTQ files using bcl2fastq Conversion Software version v2.20.0.422 (Illumina) [17].

#### 2.12.3. Raw Data Processing and Quality Control

Raw data processing was conducted by use of nfcore/rnaseq (version 1.4.2) which is a bioinformatics best-practice analysis pipeline used for RNA sequencing data at the National Genomics Infrastructure at SciLifeLab, Stockholm, Sweden [17]. The pipeline uses Nextflow, a bioinformatics workflow tool. It preprocesses raw data from FASTQ inputs, aligns the reads, and performs extensive quality control on the results [17]. The genome reference and annotation data were taken from http://GENCODE.org (Mus musculus; GRCm38.p6; release M25).

#### 2.12.4. Normalization and Differential Expression Analysis

Normalization and differential expression analysis were performed with DESeq2 (Galaxy Tool Version 2.11.40.6; DESeq2 version 1.22.1) with default settings except for “Output normalized counts table,” “Turn off outliers replacement,” “Turn off outliers filtering,” and “Turn off independent filtering,” and all of which were set to “True” [17]. The *Ec*N infection was selected as a primary factor, whereas the donor was used as a secondary factor in DESeq2 analyses (two-factor design). The results of the DESeq2 analysis are displayed in Supplementary Table 1. DESeq2 result table was loaded into Qlucore Omics Explorer (version 3.7) software using the Wizard function for visualization via heat map.

For gene set enrichment analysis, Enrichr gene set enrichment analysis web server was utilized [18]. Visualization for enrichment analysis was performed with the Appyters [19] programmatically run from the Enrichr results page with default settings for the Enrichr library KEGG 2019 Mouse.

### 2.13. Statistical Analysis

The statistical analyses were performed using GraphPad Prism 6® software (San Diego, CA, USA). The values are plotted either directly with the means and 95% confidence intervals (CIs) or standard deviations (SDs) or as the means with 95% CIs or SDs. Before calculating the means, all technical replicates were statistically tested using the Grubbs outlier test. All means from pooled groups were statistically tested via the ROUT outlier test. The following tests were performed for data with equal variances: the data from the analysis of the effects of ZellShield® on organoid kinetics were assessed by two-way analysis of variances followed by Tukey's multiple comparisons tests, the independently plotted data from experimental and biologic replicates were analyzed by a one-way analysis of variances followed by Tukey's multiple comparisons tests, and the pooled data from the biological replicates were analyzed using an unpaired *t*-test. In addition, the control and *Ec*N-infected samples of freshly isolated and cryopreserved organoids were subjected to the following pairwise comparisons by one-way analysis of variances followed by Sidak's multiple comparisons tests: fresh Ctrl vs. fresh *Ec*N, fresh Ctrl vs. thawed Ctrl, fresh *Ec*N vs. thawed *Ec*N, and thawed Ctrl vs. thawed *Ec*N. A *P* value of <0.05 was defined as significant (∗) for all experiments with the following grading: ^∗∗^*P* < 0.01, ^∗∗∗^*P* < 0.001, and ^∗∗∗∗^*P* < 0.0001.

## 3. Results

### 3.1. ZellShield® Does Not Affect Gene Expression in Colonoids

Due to practical reasons such as easier handling, the culturing of cell lines using antibiotics is a common practice. Because most conventional cell lines are derived from tumors and therefore do not properly recapitulate the physiological state, the possible side effects of antibiotics can be neglected in most studies. For primary cells collected from a nonsterile environment such as the gut, it is important to avoid the overgrowth of bacteria or fungi. Thus, the use of antibiotics is crucial for achieving and maintaining a sterile environment. However, it is commonly known that the microbial flora shapes and maintains, for example, a strong intestinal barrier [20, 21]. Therefore, its removal due to antibiotic administration could alter the physiology of primary cells and their reaction to environmental stimuli, for example, when conducting infection experiments. Additionally, antibiotics could have a direct impact on gene expression levels; thus, omitting them might have an impact on the experimental outcome.

**Figure 1:**
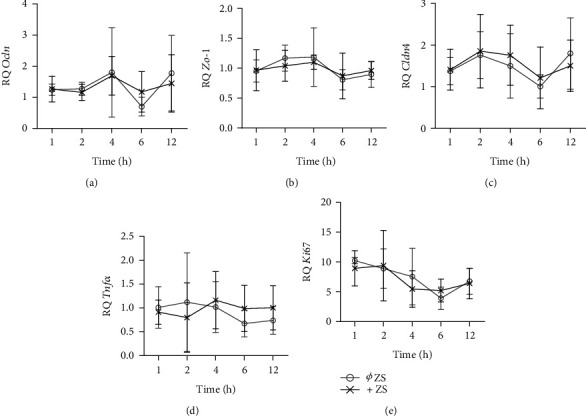
ZellShield® does not affect organoid kinetics. Comparison of the gene expression levels in colonoids over the course of 12 hours after the administration of fresh medium with (+ ZS) and without (Ø ZS) ZellShield® (each: *n* = 5). The graphs show the means with SDs. ^∗^*P* < 0.05, ^∗∗^*P* < 0.01, ^∗∗∗^*P* < 0.001, and ^∗∗∗∗^*P* < 0.0001. (a) Relative *Ocln* expression, ANOVA (*F* (9, 41) = 0.9771; *P* = 0.4728). (b) Relative *Zo-1* expression, ANOVA (*F* (9, 42) = 1.088; *P* = 0.3915). (c) Relative *Cldn4* expression, ANOVA (*F* (9, 42) = 0.9054; *P* = 0.5295). (d) Relative *Tnfα* expression, ANOVA (*F* (9, 42) = 0.4397; *P* = 0.9055). (e) Relative *Ki67* expression, ANOVA (*F* (9, 41) = 1.946; *P* = 0.0719).

To analyze possible side effects of antibiotics on colonoids, we performed a kinetic experiment with controlled addition (+ ZS) and removal (Ø ZS) of ZellShield®, a defined purchasable mix of antibiotics/antimycotics, over the course of 12 hours (h) with a sample collection 1, 2, 4, 6, and 12 hours after media administration (Figure 1). Gene expression levels of the tight junction proteins *Cldn4* (claudin 4), *Ocln* (occludin), and *Zo-1* (zonula occludens-1; tight junction protein 1) and the cytokine *Tnfα* did not differ between the two conditions and were mostly stably expressed over time with a rather high standard deviation for *Cldn4*, *Ocln*, and *Tnfα*. The proliferation marker *Ki67* was also equally expressed in both conditions, but as expected, expression slowly decreased over time. This is consistent with our earlier findings [15] and represents the consumption of fresh media. In summary, ZellShield® seemed to have no effect on tight junction expression, proliferation, or induction of *Tnfα* in colonoids. However, to minimize effects related to medium changes and to acclimate organoids, fresh medium should be administered 12-16 hours prior to all experiments.

### 3.2. Experimental Data Are Predominantly Affected by Interexperimental Differences

To analyze the technical and interexperimental reliability, as well as the biological variability among organoids, we measured the acute effects of infection with *E. coli* Nissle 1917 (*Ec*N) on gene expression in colonoids. We thus performed five independent infection experiments, hereafter referred to as experimental replicates. For each experiment, we generated three different organoid lines (biological replicates) and prepared three technical replicates of each control and *Ec*N-infected sample from each organoid line. We wanted to analyze the reproducibility of our data and which type of replicate (technical, biological, and experimental) has the highest impact on the experimental outcome.

**Figure 2:**
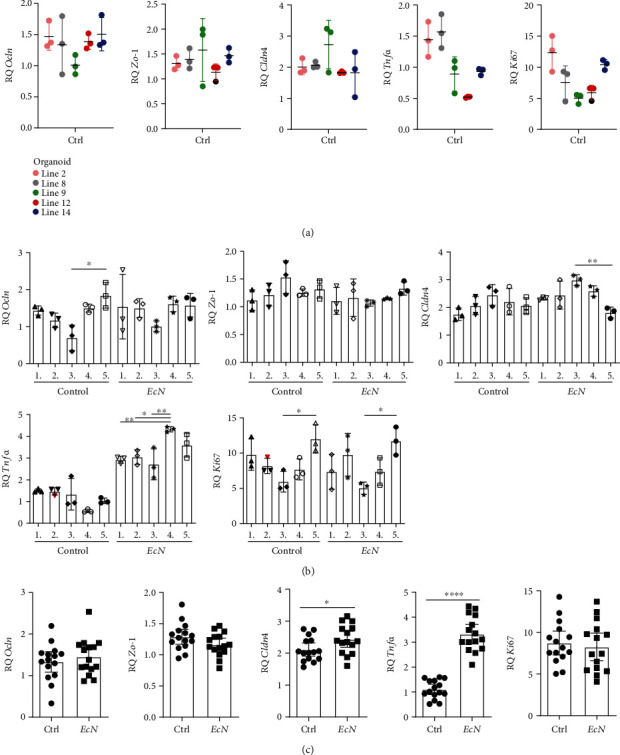
Experimental data are predominantly affected by interexperimental differences. The technical and experimental reproducibility and the biological variability in colonoids were compared using the EcN infection model. ^∗^*P* < 0.05, ^∗∗^*P* < 0.01, ^∗∗∗^*P* < 0.001, and ^∗∗∗∗^*P* < 0.0001. (a) Technical replicates. The graphs plot individual values of five representative organoid lines with the technical replicate mean and SD values; the outliers, as identified using the Grubbs outlier test, are shown as black dots. (b) Experimental and biological replicates. The graphs plot five independent experiments with the technical replicate means from three different organoid lines (biological replicates) per experiment plus the overall mean and SD per experiment; the outliers, as identified using the Grubbs outlier test, are shown as red triangles; ANOVA: Ocln (*F* (9, 20) = 2.609, *P* = 0.0355), Zo-1 (*F* (9, 20) = 1.538, *P* = 0.2019), Cldn4 (*F* (9, 20) = 4.227, *P* = 0.0035), Tnf*α* (*F* (9, 20) = 29.69, *P* < 0.0001), and Ki67 (*F* (9, 20) = 4.150, *P* = 0.0039). (c) Pooled data. The graphs plot the technical replicate means of all biological replicates (*n* = 15) from all independent experiments plus the overall mean and 95% CI.

First, we examined the clustering of the technical replicates (Figure 2(a); representative results from the control samples of one organoid line from each experiment are shown). We noted four different clustering patterns: most technical replicates clustered closely together with a rather small SD and no visual and statistically significant outlier, as observed for, for example, *Cldn4* expression in organoid lines no. 2 and 8. Other technical replicates clustered evenly apart from one another with the mean laying around the middle value, as was observed for, e.g., *Tnfα* expression in organoid lines no. 2 and 8. Few replicate groups had visual outliers but no statistical relevance, as was detected for, for example, *Zo-1* and *Cldn4* expression in organoid line no. 9. Only two technical replicate groups had statistically relevant outliers (shown as black dots), namely, *Zo-1* and *Ki67* expression in organoid line 12, and these groups were later omitted from the mean calculation. Overall, the analysis of all technical samples revealed an acceptable statistical outlier frequency of at most 2.15% per gene (maximum of two outliers out of 93 individual values per gene). Therefore, our technical replicates were rather reliable and had only a low impact on the experimental outcome.

In the next step, we analyzed the clustering of biological replicates per experiment (Figure 2(b)). We observed a clustering pattern similar to the four different patterns found for the technical replicates, but in general, the biological replicates within an experiment clustered rather closely together. Statistically, only two relevant outliers (shown as red triangles) within a biological replicate group were detected for all the genes: *Tnfα* expression in organoid line 3 and *Ki67* expression in organoid line 4. This finding equates to an overall statistical outlier frequency of at most 3.33% per gene (max. 1 out of 30 mean values per gene). After pooling the data, including the two previously mentioned outliers (Figure 2(c)), no statistically significant outliers could be detected. Therefore, the sole biological variability had a rather low impact on our data.

In the final step, we further analyzed the effects of experimental replicates on variability (Figure 2(b)) and detected several significant interexperimental differences by the ANOVA: the *Ocln* gene expression levels differed significantly between two control groups (Ctrl 3 and 5, *P* = 0.0239). In addition, the *Cldn4* expression levels were significantly different between two *Ec*N-infected groups (*Ec*N 3 and 5, *P* = 0.0054). Additionally, after *Ec*N infection, the *Tnfα* gene expression level in one experiment was significantly higher than that in three other experiments [*Ec*N 4 and *Ec*N 1 (*P* = 0.0089), 2 (*P* = 0.0201), and 3 (*P* = 0.0021)]. Although several significant differences were detected between the experiments, it is important to mention that the increasing or decreasing trends in gene expression between the control and infected samples were mostly the same in the independent experiments. For example, the *Ki67* gene expression levels differed significantly between both control samples (*P* = 0.0297) and both *Ec*N-infected samples (*P* = 0.0133) of the same experiments (nos. 3 and 5). This trend was also visually observed for other expression patterns showing nonsignificant differences. However, experimental replicates had a higher impact on our data than technical and biological replicates.

Focusing again on the pooled data (Figure 2(c)) and the overall experimental outcome, we measured a significant increase in *Cldn4* gene expression after *Ec*N infection (*P* = 0.0439), but this finding could not be detected in the single experiments, presumably due to the rather low effect. In contrast, the significant increase in *Tnfα* expression after *Ec*N infection (*P* < 0.0001) was also observed in the independent experiments (Figure 2(b); the following significant differences are not shown in the graph: comparison of Ctrl and *Ec*N in Exps. 1 to 5: *P* = 0.0073, *P* = 0.0026, *P* = 0.0098, *P* < 0.0001, and *P* < 0.0001, respectively), but these mostly exhibited a lower *P* value than that obtained for the pooled data. The gene expression levels of the tight junction proteins *Ocln* (*P* = 0.4695) and *Zo-1* (*P* = 0.1021) and the proliferation marker *Ki67* (*P* = 0.6469) did not differ between the control and *Ec*N-infected samples (Figure 2(c)). Taken together, these results show that *Ec*N has an impact on the gene expression levels of the tight junction protein *Cldn4* and on the induction of *Tnfα*.

### 3.3. Cryopreserved Organoids Show Attenuated Responses in *Ec*N Infection Experiments

**Figure 3:**
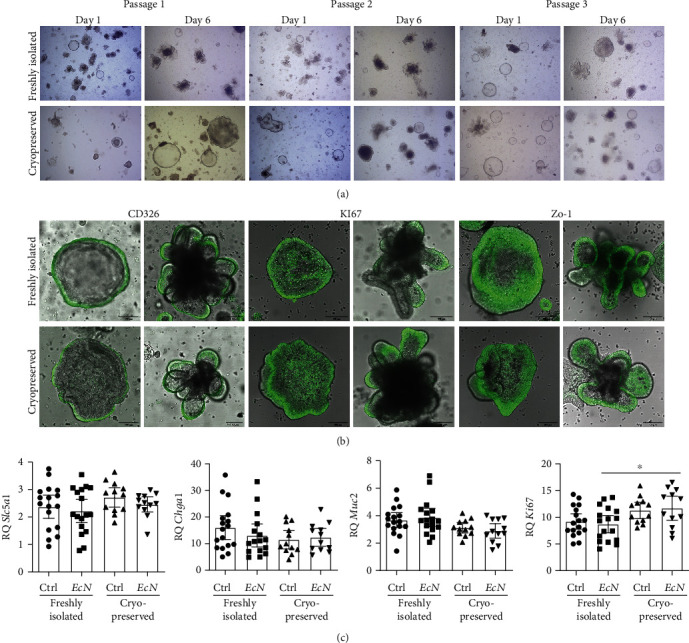
Characterization of freshly isolated and cryopreserved colonoids during growth and after *Ec*N infection. (a) Representative light phase-contrast images. (b) Immunofluorescent staining for epithelial cell adhesion molecule (CD326), KI67, and tight junction protein 1 (ZO-1). (c) The gene expression levels in freshly isolated colonoids (*n* = 17) and cryopreserved and thawed organoids (*n* = 12) of the same passage were compared before and after infection with *Ec*N. The graphs plot the pooled technical replicate means of biological replicates plus the overall mean and 95% CI. ^∗^*P* < 0.05, ^∗∗^*P* < 0.01, ^∗∗∗^*P* < 0.001, and ^∗∗∗∗^*P* < 0.0001. Relative *Slc5a1* expression (ANOVA: *F* (3, 54) = 1.179; *P* = 0.3264). Relative *Chga1* expression (ANOVA: *F* (3, 53) = 1.104; *P* = 0.3555). Relative *Muc2* expression (ANOVA: *F* (3, 54) = 2.932; *P* = 0.0416). Relative *Ki67* expression (ANOVA: *F* (3, 54) = 3.435; *P* = 0.0231).

**Figure 4:**
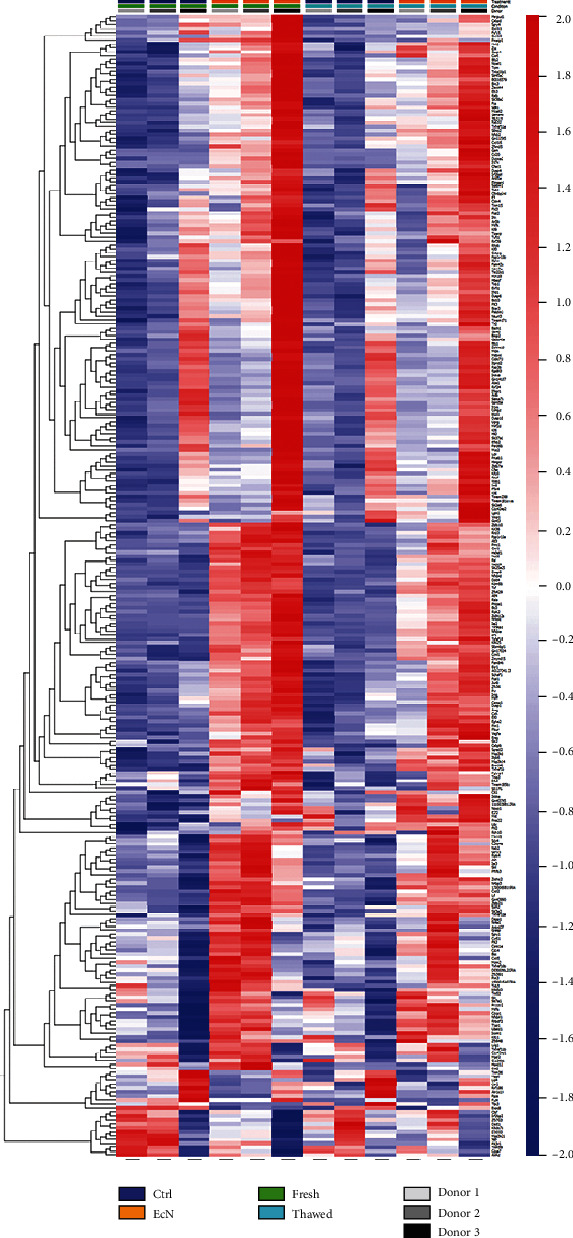
Fresh and cryopreserved organoids exhibit a similar gene expression profile in response to *Ec*N injection. Heat map displaying significantly (adjusted *P* value < 0.01) differentially expressed genes detected between *Ec*N and control samples in fresh colonoids. Besides the normalized and log transformed expression levels of the samples originating fresh organoids, the heat map includes in an analogous manner the samples of thawed colonoids.

**Figure 5:**
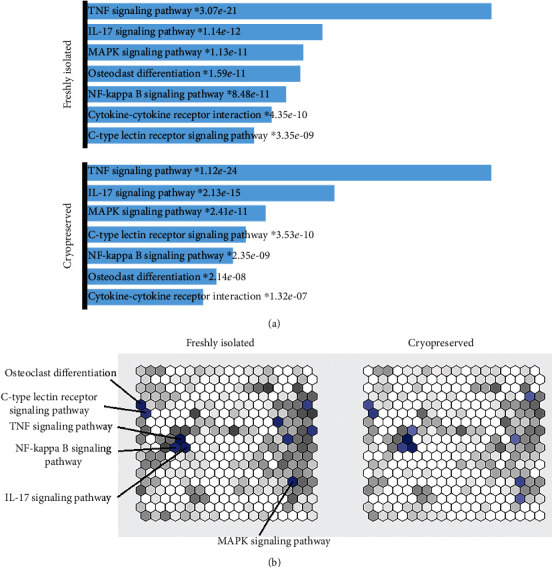
Gene set enrichment analysis. Significantly (adjusted *P* value < 0.01) upregulated genes in *Ec*N compared to control samples originating from fresh and cryopreserved colonoids, respectively, were examined for their similarity to gene sets of the KEGG 2019 Mouse library via Enrichr gene set enrichment analysis web server. (a) Top enriched terms in the KEGG 2019 Mouse library, with *P* values. Asterisk symbolizes the term has an adjusted *P* value < 0.05. (b) Each hexagon represents one gene set from the KEGG 2019 Mouse library. The brighter the blue color is, the more similar and, therefore, significant the specific gene set. Hexagons that are grouped together represent similar gene sets.

**Figure 6:**
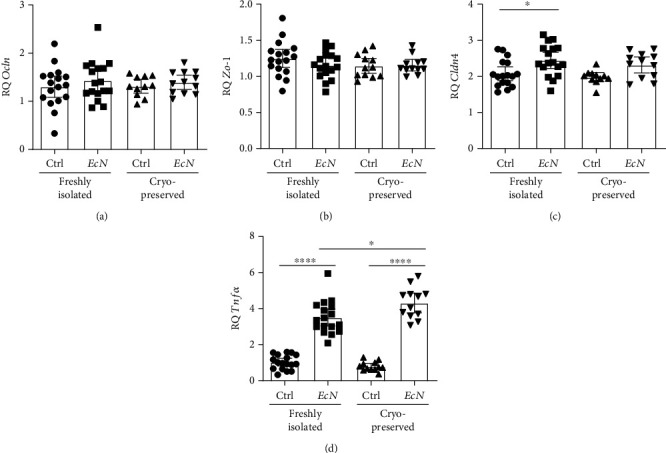
Cryopreservation affects gene expression levels in colonoids after *Ec*N infection. The gene expression levels in freshly isolated colonoids (*n* = 17) and cryopreserved and thawed organoids (*n* = 12) of the same passage after infection with *Ec*N were compared. The graphs plot the pooled technical replicate means of biological replicates plus the overall mean and 95% CI. ^∗^*P* < 0.05, ^∗∗^*P* < 0.01, ^∗∗∗^*P* < 0.001, and ^∗∗∗∗^*P* < 0.0001. (a) Relative *Ocln* expression (ANOVA: *F* (3, 53) = 0.4720; *P* = 0.7031). (b) Relative *Zo-1* expression (ANOVA: *F* (3, 54) = 1.010; *P* = 0.3953). (c) Relative *Cldn4* expression (ANOVA: *F* (3, 53) = 4.962; *P* = 0.0041). (d) Relative *Tnfα* expression (ANOVA: *F* (3, 54) = 91.30; *P* < 0.0001).

One of the major advantages of the organoid system is the ability to propagate organoids shortly after isolation and subsequently cryopreserve them until further use [6], similarly to regular nonprimary cell lines. However, it is commonly known that the freezing and thawing of cells is an invasive treatment that can alter not only the cell viability but also other parameters within a cell, such as gene expression patterns. To analyze the possible effects of cryopreservation on primary organoid cell culture and on the outcome of infection experiments, we first compared the morphology of freshly isolated colonoids with cryopreserved and subsequently thawed colonoids (hereafter referred to as cryopreserved colonoids, Figures 3(a) and 3(b)); then, we performed infection experiments on both colonoid types in passage 3. We compared the acute effects of *Ec*N infection on gene expression levels and different signaling pathways (Figures 3(c) and 4–6). For a direct comparison within the same biological replicates, freshly isolated colonoids were also used for cryopreservation, thawed, and then infected together with new freshly isolated colonoids of the same passage number. During passage 1, more and bigger colonosphere structures were observed in the cryopreserved culture compared to freshly isolated organoids (Figure 3(a)). No differences were detectable in passages 2 and 3. Immunohistological staining for CD326, a marker for epithelial cells, showed a positive signal in the outer cell layer of colonospheres and organoids (Figure 3(b)). High amounts of KI67-positive cells were found throughout the whole epithelium of all colonospheres, and positive cells in mature colonoids were located at the base and sides of the intestinal crypts. Colonospheres as well as organoids were positive for the intracellular TJ protein ZO-1 (Figure 3(b)). In addition, epithelial cell subtypes such as enterocytes (*Slc5a1*), enteroendocrine cells (*Chga1*), and goblet cells (*Muc2*) were analyzed using qPCR before and after *Ec*N infection. All analyzed genes did not differ between freshly isolated and cryopreserved colonoids (Figure 3(c)). After *Ec*N infection, *Ki67* expression was significantly higher in the cryopreserved organoids (*P* = 0.0425) and tended to be higher in the control cryopreserved organoids than in freshly isolated colonoids.

Furthermore, we performed RNA sequencing and pathway analysis from freshly isolated and cryopreserved colonoids after *Ec*N infection. Although donor and interexperimental-specific differences were detectable, overall, all genes which were significantly (adjusted *P* value < 0.01; Supplementary Table 2) differentially expressed in *Ec*N samples in fresh colonoids compared to control counterparts displayed a comparable expression pattern in thawed organoids (Figure 4). The change in gene expression levels between *Ec*N and control samples was attenuated in cryopreserved colonoids compared to fresh ones, though. While in fresh colonoids, the significantly differentially expressed genes (DEGs) amounted up to 290; in cryopreserved colonoids, only 140 significantly DEGs were detected between *Ec*N and control samples (Supplementary Table 3). Notably, almost all of those 140 significantly DEGs were also found among the 290 genes in the group of fresh organoids without indication for any additional effect of the thawing process on *Ec*N treatment outcome.

Correspondingly, both fresh and cryopreserved colonoids experienced gene upregulation in, overall, the same pathways or biological processes in response to *Ec*N infection (Figure 5). Upregulated genes were especially associated with the TNF, IL-17, MAPK, or NF-kappa B signaling pathway (Figure 5(a)). Although the same signaling pathways were activated in fresh and cryopreserved organoids after *Ec*N infection, the association with each gene set, except the TNF and IL-17 signaling pathway (Figure 5(a)), was more significant for the fresh colonoids due to the higher number of significantly upregulated genes (Figure 5(b)).

In addition, the relative gene expression of the tight junction genes *Ocln*, *Zo-1*, and *Cldn4* before or after infection did not significantly differ between the two types of colonoids (Figure 6). However, the *Cldn4* expression levels were significantly elevated in fresh organoids after *Ec*N infection (*P* = 0.0182), whereas no significant differences were detected by ANOVA in the cryopreserved organoids (*P* = 0.1144). But a direct comparison using an unpaired *t*-test showed a significant difference between the control and *Ec*N-infected samples of cryopreserved colonoids (*P* = 0.0124). In addition, *Tnfα* expression was significantly upregulated in both types of colonoids (both *P* < 0.0001) in response to bacterial stimulation. However, after *Ec*N infection, significantly higher expression was detected in the cryopreserved organoids than in the fresh colonoids (*P* = 0.0110). Together, these results indicate that strong effects can be easily observed in cryopreserved colonoids, whereas smaller effects might remain undetected.

## 4. Discussion

As often stated in various articles, comments, and reviews, standardization techniques have been needed in the field of organoid research for a longer period [5–7, 22]. Because every laboratory uses its own methods for conducting experiments and uses different tissue/cell sources, among other variations, the recreation of results and their transferability to other labs are extremely difficult. According to our early personal experience with organoid culture, even the reproducibility of our results proved to be challenging, and we therefore developed several methods for standardizing our colonoids. Here, we provide some insights into these techniques and aim to answer several questions we encountered during our colonoid research over the last few years.

As stated previously, the commonly used cell lines and colonoids are usually cultured in the presence of antibiotics and/or antimycotics, such as ZellShield®. A culture without these agents would lead to severe infection and overgrowth of the endogenous microbiota and fungi that are naturally present in the donor tissue and cannot be mechanically removed during crypt isolation. Another option is the use of a tissue derived from germ-free mice, but germ-free animals often pose other challenges and do not recapitulate the physiological state of the gut. For example, germ-free mice have a weakened intestinal barrier, reduced metabolic rates, and an enlarged cecum because their body has to cope with the lack of digestive microbiota [23]. In addition, biopsy samples for the preparation of human intestinal epithelial organoids cannot be obtained from a germ-free individual; therefore, antibiotic administration is necessary. However, it is known that antibiotic treatment can alter the gene expression levels of epithelial cells and immune cells [24–27], and the sudden lack of antibiotics might be responsible for any effects detected in these cells. Therefore, we analyzed the impact of ZellShield® removal on the gene expression levels of the tight junction proteins *Ocln*, *Zo-1*, and *Cldn4* and the proliferation marker *Ki67* and the induction of the proinflammatory cytokine *Tnfα* in colonoids over the course of 12 hours. We did not detect any differences between culture with and without ZellShield® and concluded that ZellShield® has no effect on the expression of the analyzed genes in colonoids. However, this study and our previously published work [15] revealed that the administration of fresh medium has a direct impact on colonoid gene expression. It has been shown that standardized and improved culture conditions [28, 29] together with well-timed media administration will result in optimized experimental settings [15]. Therefore, we recommend the administration of fresh medium 12-16 hours before performing any experiments with new medium to minimize the effects related to medium changes. For optimal results, specific analyses for each case should be performed.

As mentioned previously, at the beginning of our work with intestinal organoids a few years ago, we experienced a lack of reproducibility in our experimental data. We considered factors that might influence the outcome of experiments with organoids, and according to good scientific practice, a well-planned study design is an important factor for obtaining reproducible data. Therefore, we were interested in the reliability of the experimental data obtained from technical and experimental replicates of colonoids and the extent to which biological variability might influence these data. The last factor, biological variability, is also important because the organoid system is aimed at replacing animal experiments and reducing the number of animals used according to the 3Rs. As a model setup for this study, we used an infection experiment with the probiotic bacterium *E. coli* Nissle 1917 that we previously established in our colonoids [15] and measured the gene expression levels of the abovementioned markers. Our observations revealed that both technical and biological replicates were rather reliable with few statistical outliers and thus had a rather low impact on our data. In contrast, experimental replicates exhibited more interexperimental differences and therefore had a higher impact on the produced data. These findings are consistent with those obtained by Pamies et al. [22], who state that standardizing organoids is highly demanding due to their complexity, which “can be associated with variability between individual […] experiments, thus affecting reproducibility of […] quality and functionality and hence any downstream readouts.” Overall, we conclude from our analyses that it is more important to conduct an experiment several times instead of adding many technical and biological replicates to only one or two experiments. The exact quantity of these parameters also depends on the expected outcome and statistical power, which should be measured in preliminary tests. In our previously published work [15], we standardized our data from infected/stimulated samples to the corresponding controls to further account for interexperimental differences. The rather low impact of biological variability on gene expression levels indicates that reducing the numbers of animals used for the generation of organoids of animal origin is possible. However, Voelkl et al. [30] note that it is also important to introduce some heterogenization to achieve increased reliability and to avoid idiosyncratic results, for example, from only using mice of the same age or the same genetic/microbial background. Regarding the observed effects obtained after pooling our data, the increases in the gene expression levels of the tight junction protein *Cldn4* and the proinflammatory cytokine *Tnfα* are consistent with our prior experimental results and might suggest a positive probiotic effect of *Ec*N on the epithelium, which might involve enhancing the barrier through the upregulation of tight junction components and the recruitment of immune cells via cytokine induction [15]. In tubular cells, *Tnfα* increases the gene expression and surface levels of *Cldn4* and thereby contributes to an increase in the transepithelial resistance [31]. Other in vitro studies with IECs have also shown that the response to *Ec*N stimulation and other probiotic bacteria is transiently proinflammatory [32, 33]. Therefore, our research group suggested that the upregulation of cytokines such as *Tnfα* might be part of the probiotic effect of *Ec*N [34]. Furthermore, Yan et al. [35, 36] reported that *Tnfα* is responsible for the activation of both pro- and anti-inflammatory signaling pathways and that their balance is crucial in IBD.

Again, to reduce the use of experimental animals and for the storage of patient-derived organoid cultures, using cryopreserved organoids is a major advantage of the whole organoid system. However, Pamies et al. [22] note that the cryopreservation of organoids (among others) is more complex than that of standard cell culture; hence, the maintenance of their functionality has to be ensured. To the best of our knowledge, the experimental reliability of cryopreserved murine organoids has not yet been investigated, and in our early experience with cryopreserved and then thawed organoids, we noticed that these showed a different growth pattern during the first passage compared with freshly isolated organoids. Formerly cryopreserved colonoid cultures appear to have a higher quantity of premature spheroid structures and exhibit delayed development. Therefore, we wanted to analyze their experimental behavior compared with that of freshly isolated colonoids in early passages. For this purpose, we also used *Ec*N infection as our experimental setup. To heed the European directive 2010/63/EU [13] to reduce animal numbers, we cryopreserved our freshly isolated colonoids used in our other experiments and thawed most volumes for comparison. In addition, most of the data from the freshly isolated organoids were obtained from the experiments with “different replicates,” which were conducted simultaneously. Hence, the same colonoids were used for both states, which also increase the comparability of the results. Further comparison of the cryopreserved and freshly isolated organoids showed no differences in cell subtype composition but a higher expression of *Ki67* in the cryopreserved colonoids, which hints that these are found at a presumably more premature state than fresh colonoids at the same passage. For additional analysis, we performed RNA sequencing analysis and revealed more DEGs in *Ec*N-infected freshly isolated colonoids compared to cryopreserved colonoids. However, cryopreserved organoids displayed a comparable expression pattern. As previously observed, the gene expression levels of the tight junction marker *Cldn4* and the cytokine *Tnfα* were significantly increased in the *Ec*N-infected samples of both types, although lower upregulation of *Cldn4* expression was observed in the cryopreserved colonoids. This different cell status might influence the response to bacterial challenge and might also be responsible for the significantly higher expression of the cytokine *Tnfα* observed in the cryopreserved compared with the freshly isolated colonoids. For example, during the tumor progression of gastroenteropancreatic neuroendocrine neoplasms, the expression of *Tnfα* is positively correlated with high proliferation rates, as indicated by *Ki67* expression [37]. Taken together, these results indicate that strong effects, such as the increase in *Tnfα* expression, can be easily detected in cryopreserved and possibly more premature colonoids, whereas smaller effects, such as the upregulation of *Cldn4*, might be more easily detected in freshly isolated, more mature colonoids. Therefore, the developmental characteristics of organoids should be tested before to their use in experiments to analyze which passage is optimal for experimental usage. Whether cryopreserved organoids also show different behaviors at older passages remains to be analyzed, but another study using bovine colonoids showed that formerly in-plate *in situ* cryopreserved colonoids showed similar growth rates to unfrozen colonoids of the same passage and found no significant increase in cytotoxic sensitivity to staurosporine after *in situ* freeze-thawing [38]. Other studies regarding aging in intestinal epithelial organoids describe organoid culture as an aging system similar to the *in vivo* state [39–42]. Therefore, it is likely that cryopreserved murine colonoids also mature over time and can be reliably used for experiments, which would enable a further reduction in animal numbers according to the 3Rs. For this purpose, another possibility might be the cryopreservation of whole tissue samples using the DMSO slow-freeze technique for later organoid generation, as was previously described for tumor-derived organoids by Walsh et al. [43]. These researchers observed similar *Ki67* expression and a matching drug response in organoids generated from a fresh and DMSO frozen tumor tissue a few days after generation.

In general, studies involving organoids should be carefully designed such that the lowest number of animals is used for the highest number of experiments. Of course, this poses a challenge related to the planning and interweaving of all experiments being conducted, but the same stipulation has to be considered in official animal experiments.

## 5. Conclusions

In conclusion, cultivation with or without ZellShield® had no impact on the analyzed genes of interest. Regarding good scientific practice, we showed that the experimental outcome is predominantly influenced by interexperimental differences and that the technical and biological variabilities are rather low. In addition, the cryopreservation of organoids might also influence the experimental outcome due to a possibly premature character of organoids at early passages and their higher proinflammatory response to bacterial stimulation. Therefore, testing the growth characteristics of organoids prior to their use in experiments would be recommended and will aid further standardization of organoid culture.

## Data Availability

All protocols used or data generated in this study are available from the corresponding author on reasonable request.

## Supplementary Materials

Supplementary Materials Table 1: DESeq2 result table. Table 2: Significantly different gene expression from *Ec*N samples in fresh colonoids compared to control counterparts. Table 3: Significantly differentially expressed genes between *Ec*N and control samples.
